# Supplementary material for: Imaging the state-to-state charge-transfer dynamics between the spin-orbit excited Ar+(2P1/2) ion and N2
Source: Nat Commun. 2024 Feb 2;15:1001. doi: 10.1038/s41467-024-45344-6 (PMC11258295; doi:10.1038/s41467-024-45344-6)
Supplement: Supplementary file 1 — Supplementary Information [file 41467_2024_45344_MOESM1_ESM.pdf]

Supplementary Information

Imaging the state-to-state charge-transfer dynamics between the spin-orbit excited $Ar^+(^2P_{1/2})$ ion and N_2

Guodong Zhang[1,2,#], Dandan Lu[3,#], Hua Guo[3,*], and Hong Gao[1,2,*]

[1] Beijing National Laboratory for Molecular Sciences (BNLMS), Institute of Chemistry, Chinese Academy of Sciences, Beijing 100190, China

[2] University of Chinese Academy of Sciences, Beijing 100049, China

[3] Department of Chemistry and Chemical Biology, Center for Computational Chemistry, University of New Mexico, Albuquerque, NM 87131, USA

[*]E-mail: hguo@unm.edu (Hua Guo), honggao2017@iccas.ac.cn (Hong Gao)

[#]: these authors contributed equally

Supplementary Note 1. Collision energy dependence of the charge-transfer reaction

The central slice images of the three-dimensional N_2^+ velocity distribution for the charge-transfer reaction $Ar^+(^2P_{1/2})+N_2 \rightarrow Ar+N_2^+(v', J')$ at the COM collision energies of 1.10 eV, 0.83 eV and 0.57 eV are presented in Supplementary Fig. 1(a), (b) and (c), respectively. The corresponding experimentally measured and theoretically calculated product angular distributions in the v'=1 and 2 levels at the three collision energies are presented in Supplementary Fig. 2. The calculated N_2^+ product rotational distributions and the correlation contour maps between the N_2^+ product rotational distribution and the scattering angle in the v'=1 and v'=2 levels at the three collision energies are presented in Supplementary Fig. 3.

Supplementary Note 2. Estimation of the N_2^+ product vibrational distributions

As discussed in the main text, an accurate determination of the product vibrational populations from the scattering images are not feasible, mainly because the individual vibrational levels are not completely resolved from each other. Furthermore, the degree of product rotational excitation strongly depends on the vibrational level and the angular range that the products are scattered into, thus there is not a definite way to divide the images into areas that belong to specific product vibrational levels. Nevertheless, we have made an estimation by fitting the integrated product velocity distributions with Gaussian profiles. Each product vibrational level is represented by a Gaussian peak, and the peak positions are calculated according to the energy conservation law. However, the widths and positions of the vibrational peaks are strongly affected by the angular dependent rotational excitations as discussed in the main text. In the fitting process, the peak heights are set as free parameters, while the peak widths are adjusted to be about the same for all vibrational levels, and the peak positions are also adjusted slightly to have the best agreement with the experimental curves. A typical fitting result for the scattering image at the COM collision energy of 1.58 eV is presented in Supplementary Fig. 4. The underneath areas of the Gaussian profiles are then calculated as the relative population ratios of the corresponding vibrational levels.

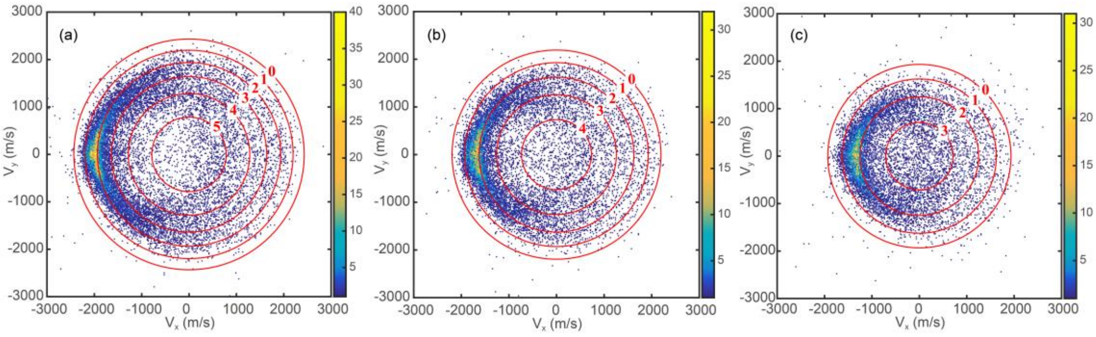

Supplementary Fig. 1 | Product imaging at lower collision energies. The central slice images of the three-dimensional N_2^+ velocity distribution for the charge-transfer process between the spin-orbit excited $Ar^+(^2P_{1/2})$ ion and N_2 at the COM collision energies of (a) 1.10 eV, (b) 0.83 eV and (c) 0.57 eV. The kinematic cutoffs for each vibrational levels of N_2^+ considering the anharmonic corrections are indicated by the red concentric rings.

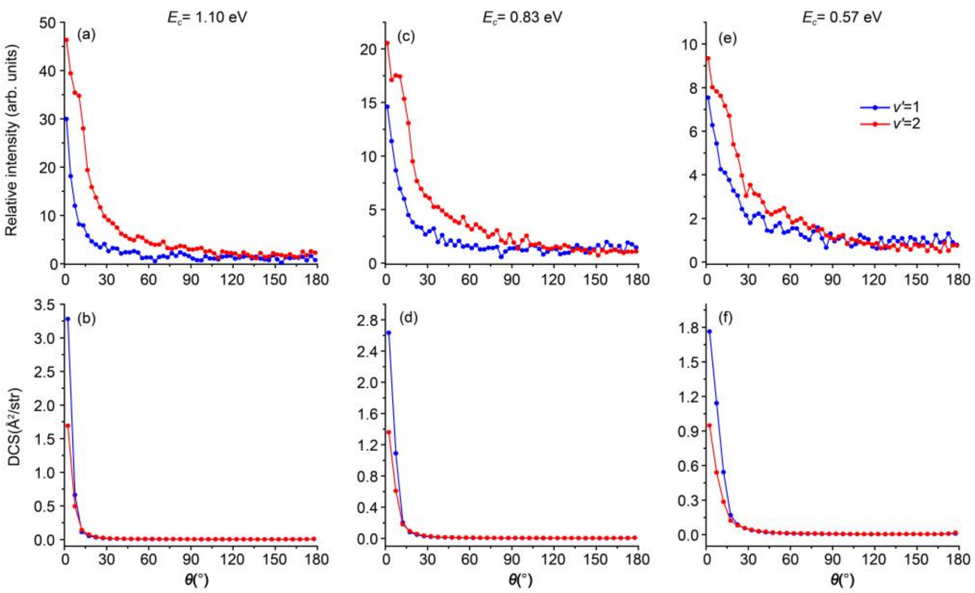

Supplementary Fig. 2 | Product angular distributions at lower collision energies. Experimentally measured angular distributions of the N_2^+ product in the v'=1 (blue) and 2 (red) levels at the COM collision energies of (a) 1.10 eV, (c) 0.83 eV and (e) 0.57 eV for the charge-transfer reaction $Ar^+(^2P_{1/2})+N_2 \rightarrow Ar+N_2^+(v', J')$; (b) Calculated N_2^+ product angular distributions by the trajectory surface hopping method at the COM collision energies of (b) 1.10 eV, (d) 0.83 eV and (f) 0.57 eV, respectively.

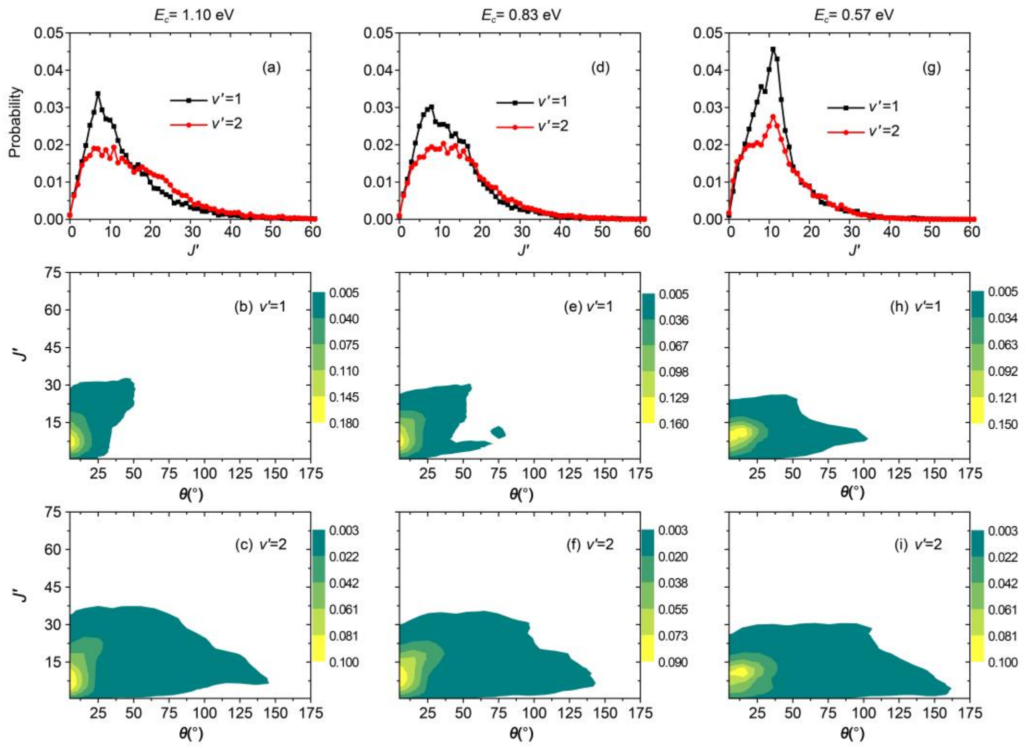

Supplementary Fig. 3 | Product rotational distributions and their correlations with the scattering angles at lower collision energies. The N_2^+ product rotational distributions in the v'=1 (black) and 2 (red) levels for the charge-transfer reaction $Ar^+(^2P_{1/2})+N_2 \rightarrow Ar+N_2^+(v', J')$ at the COM collision energies of (a) 1.10 eV, (d) 0.83 eV and (g) 0.57 eV calculated by the trajectory surface hopping method. The calculated correlation contour maps between the N_2^+ product rotational distribution and the scattering angle for v'=1 and v'=2 levels are presented in (b, c), (e, f) and (h, i) for the COM collision energies of 1.10 eV, 0.83 eV and 0.57 eV, respectively.

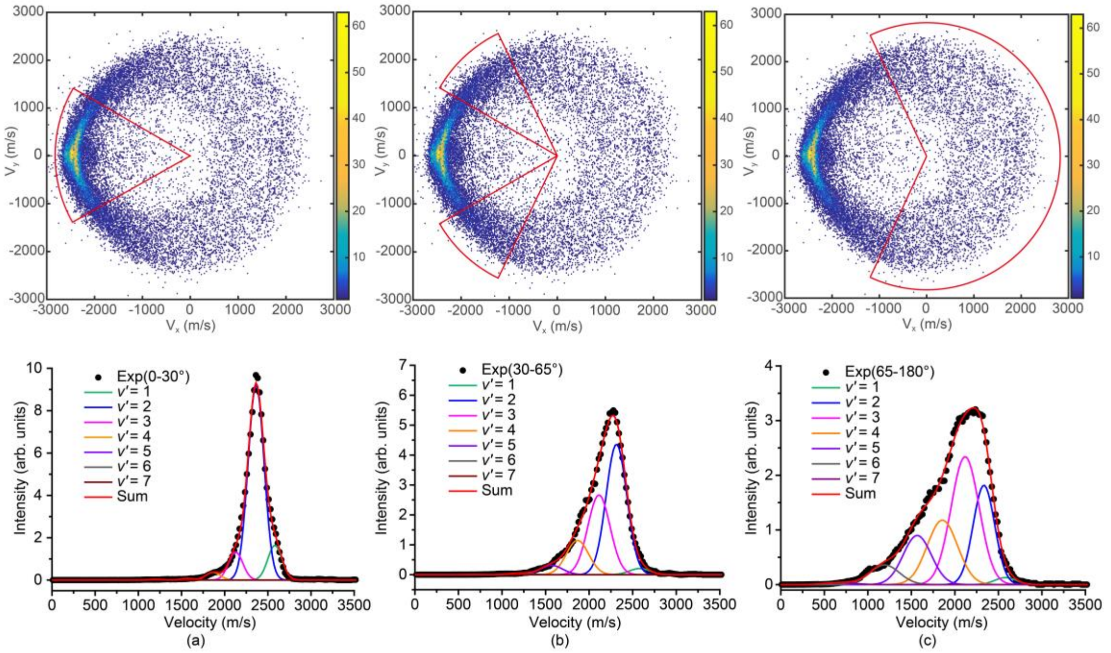

Supplementary Fig. 4 | Estimation of the N_2^+ vibrational populations. Estimation of the N_2^+ vibrational populations in the three angular ranges: (a) 0-30°; (b) 30-65°; (c) 65-180° for the charge-transfer reaction $Ar^+(^2P_{1/2})+N_2 \rightarrow Ar+N_2^+(v', J')$ at the COM collision energy of 1.58 eV.
